# Dynamic Bcl-xL (S49) and (S62) Phosphorylation/Dephosphorylation during Mitosis Prevents Chromosome Instability and Aneuploidy in Normal Human Diploid Fibroblasts

**DOI:** 10.1371/journal.pone.0159091

**Published:** 2016-07-11

**Authors:** Prasamit Saurav Baruah, Myriam Beauchemin, Josée Hébert, Richard Bertrand

**Affiliations:** 1 Centre de recherche, Centre hospitalier de l’Université de Montréal (CRCHUM), Montreal, Québec, Canada; 2 Institut du Cancer de Montréal, Montreal, Québec, Canada; 3 Quebec Leukemia Cell Bank, Centre de recherche, Hôpital Maisonneuve-Rosemont, Montreal, Québec, Canada; 4 Département de médecine, Université de Montréal, Montréal, Québec, Canada; Institut de Génétique et Développement de Rennes, FRANCE

## Abstract

Bcl-xL proteins undergo dynamic phosphorylation/dephosphorylation on Ser49 and Ser62 residues during mitosis. The expression of Bcl-xL(S49A), (S62A) and dual (S49/62A) phosphorylation mutants in tumor cells lead to severe mitotic defects associated with multipolar spindle, chromosome lagging and bridging, and micro-, bi- and multi-nucleated cells. Because the above observations were made in tumor cells which already display genomic instability, we now address the question: will similar effects occur in normal human diploid cells? We studied normal human diploid BJ foreskin fibroblast cells expressing Bcl-xL (wild type), (S49A), (S49D), (S62A), (S62D) and the dual-site (S49/62A) and (S49/62D) mutants. Cells expressing S49 and/or S62 phosphorylation mutants showed reduced kinetics of cell population doubling. These effects on cell population doubling kinetics correlated with early outbreak of senescence with no impact on the cell death rate. Senescent cells displayed typical senescence-associated phenotypes including high-level of senescence-associated β-galactosidase activity, interleukin-6 (IL-6) secretion, tumor suppressor p53 and cyclin-dependent kinase inhibitor p21Waf1/Cip1 activation as well as γH2A.X-associated nuclear chromatin foci. Fluorescence *in situ* hybridization analysis and Giemsa-banded karyotypes revealed that the expression of Bcl-xL phosphorylation mutants in normal diploid BJ cells provoked chromosome instability and aneuploidy. These findings suggest that dynamic Bcl-xL(S49) and (S62) phosphorylation/dephosphorylation cycles are important in the maintenance of chromosome integrity during mitosis in normal cells. They could impact future strategies aiming to develop and identify compounds that could target not only the anti-apoptotic domain of Bcl-xL protein, but also its mitotic domain for cancer therapy.

## Introduction

The Bcl-2 family of proteins, including Bcl-xL [1], stands out among key regulators of apoptosis, executing crucial functions and controlling whether cells will live or die during development and cellular stress [2]. Studies have revealed that members of the Bcl-2 family, in addition to their central role in apoptosis, are also involved in membrane dynamics and remodelling [3, 4], cell cycle regulation [5–12], DNA damage responses, repair and recombination [13–17], effects that are generally distinct from their function in apoptosis.

The pleiotropic functions of Bcl-xL depend at least on post-translational modifications and its sub-cellular location. Bcl-xL phosphorylation on Ser62 residues was first detected in various cancer cell lines treated with microtubule inhibitors [18–20], and later found in synchronized cells [11]. A subset of the Bcl-xL protein pool undergoes dynamic phosphorylation at Ser62 during the S and G2 phases of the cell cycle, followed by a high phosphorylation peak during the early step of mitosis [11, 12]. During cell cycle progression, Polo kinase 1 (PLK1) and mitogen-activated protein kinase 9 / c-jun N-terminal kinase 2 (MAPK9/JNK2) are major protein kinases associated with progressive phosphorylation of Bcl-xL(S62) during G2, where it accumulates in nuclear structures, including nucleoli and Cajal bodies [11].

During mitosis, Bcl-xL(S62) is strongly phosphorylated by PLK1 and MAPK14/ stress-activated protein kinase p38α (SAPKp38α) at the prophase, prometaphase and metaphase/ anaphase boundaries, with its rapid dephosphorylation at telophase and cytokinesis [12]. At mitosis, phospho-Bcl-xL(S62) localizes in centrosomes with γ-tubulin and in mitotic cytosol with some spindle-assembly checkpoint (SAC) signaling components including PLK1, BubR1 and Mad2. In taxol- and nocodazole-exposed cells, phospho-Bcl-xL(S62) also binds to Cdc20-, Mad2-, BubR1-, and Bub3-complexes, while the phosphorylation mutant Bcl-xL(S62A) does not [12].

Dynamic cell cycle-dependent Bcl-xL phosphorylation at Ser49 also has been reported. In synchronized cells, phospho-Bcl-xL(S49) appears during the S and G2 phases, whereas it disappears rapidly in early mitosis during prophase, prometaphase and metaphase, re-appearring during ongoing anaphase, telophase and cytokinesis [10]. During G2, a significant phospho-Bcl-xL(S49) protein pool accumulates in centrosomes, particularly after DNA damage-induced G2 arrest, while during telophase and cytokinesis, it is found with microtubule-associated dynein motor protein and in the mid-zone body. PLK3 is the key protein kinase involved in Bcl-xL(S49) phosphorylation [10].

Ser49 and Ser62 residues are located within the unstructured loop domain of Bcl-xL, a region generally not essential for its anti-apoptotic function [9–12, 21, 22]. Indeed, Bcl-xL's anti-apoptotic function is inherent to the BH1, BH2 and BH3 domains of the protein that create a hydrophobic pocket where the amphipathic α-helix of another BH3-containing protein can bind [23–25]. Bcl-xL proteins exert their anti-apoptotic activity by binding to and inactivating pro-apoptotic members of the family, including Bax and Bak. In contrast, a subset of Bcl-2 pro-apoptotic members (BH3-only proteins), mediate interaction with Bcl-xL and inhibit the anti-apoptotic function, thereby promoting apoptosis [26–28].

In tumor cells, expression of the phosphorylation mutants Bcl-xL(S62A), Bcl-xL(S49A) and dual Bcl-xL(S49/62A) shows anti-apoptotic properties similar to Bcl-xL wild-type (wt) protein. However, expression of the phosphorylation mutants Bcl-xL(S62A), Bcl-xL(S49A) and dual Bcl-xL(S49/62A) leads to an increased number of cells harbouring mitotic defects, as visualized by time-lapse live-cell imaging microscopy [12]. These defects include multipolar spindle, chromosome lagging and bridging, micro-, bi- and multi-nucleated cells, and cells that fail to complete mitosis [12]. Together, these observations indicate that during mitosis, Bcl-xL(S49) and (S62) phosphorylation/dephosphorylation dynamics impact on chromosome stability, mitosis resolution and cytokinesis completion.

Because the above findings occurred in tumor cells, which already display genomic instability with chromosome aberrations and aneuploidy, the present studies were performed in normal human diploid BJ fibroblast cells. BJ cells have a normal very stable diploid karyotype at population doubling up to 62, but begin to display karyotype abnormalities at population doubling of 80 at the outbreak of replicative senescence [29]. We hypothesized that although BJ cells have a normal and very stable genetics until the outbreak of replicative senescence, the expression of Bcl-xL (S49) and/or (S62) phosphorylation mutants will provoke their early chromosomal instabilty and aneuploidy.

## Materials and Methods

### Cell culture, cDNA constructs, lentivirus preparations and cell analysis

Human BJ cell lines were obtained directly from the American Type Culture Collection at population doubling of 22 with authentication, and grown at 37°C under 5% CO_2_ in Eagle's minimum essential medium (EMEM) supplemented with 10% heat-inactivated fetal bovine serum (FBS), 100 U/ml penicillin and 100 μg/ml streptomycin, respectively. Cell numbers were counted by standard hemocytometer in duplicate. All cDNA constructs were generated and subcloned in pLenti6.2 Blast DEST vector (Invitrogen Corporation, Carlsbad, CA) as described previously [10–12]. All vectors were sequenced in both orientations.

Lentiviruses were produced in 293FT cells, also obtained from Invitrogen. Lentivirus titrations were performed according to the manufacturer’s protocol and a MOI of 1 was used for infection. Transduced BJ cells with lentiviruses were grown under blasticidin (7 μg/ml) selection for 2 weeks.

In senescence-associated β-galactosidase (SA-β-gal) assays, cells were fixed in 3% formaldehyde buffered with phosphote-buffered saline (PBS) for 2–3 min, then washed with PBS. They were incubated in a staining solution containing 20 mM citrate-phosphate, pH 6.0, 150 mM NaCl, 5 mM potassium ferricyanide, 5 mM potassium ferrocyanide, 2 mM MgCl_2_ and 200 μM chromogenic substrate 5-bromo-4-chloro-3-indoyl β-D-galactopyranoside in a humidified chamber at 37°C for 24h in the dark [9]. The cells were then washed and visualized by phase contrast microscopy. Cell death was monitored by standard Hoechst 33342- and propidium iodine (PI)-staining with visualization by fluorescence microscopy. IL-6 secretion was measured using Human IL-6 ELISA Ready-Set-Go reagent set, according to the manufacturer's instructions (eBioscience Inc, SanDiego, CA).

### Protein extraction and immunoblotting

To prepare total protein, cells were extracted with lysis buffer containing 20 mM Hepes- KOH, pH 7.4, 120 mM NaCl, 1% Triton X-100, 2 mM phenylmethylsulfonyl fluoride, a cocktail of protease inhibitors (Complete^TM^, Roche Applied Science, Laval QC) and a cocktail of phosphatase inhibitors (PhosStop^TM^, Roche Applied Science). The antibodies (Abs) in this study were Bcl-xL (54H6) rabbit monoclonal Ab (mAb), Ki-67(8D5) mouse mAb, p21Waf1/Cip1(12D1) rabbit mAb, p16/INK4A rabbit polyclonal Ab (pAb) and p53(1C12) mouse mAb obtained from Cell Signaling Technology Inc. (Beverly, MA). Phospho-histone H2A.X (Ser139) (JBW301) mouse mAb were purchased from EMD Millipore Corporation (Temecula, CA), and β-actin (AC-15) mouse mAb was from Abcam Inc. (Cambridge, MA). Peroxidase-labeled secondary Ab were detected by enhanced chemiluminescence with reagent set from GE Healthcare Life Science (Mississauga, ON) or SuperSignal WestPico chemiluminescence substrates from Thermo Scientific (Rockford, IL).

### Fluorescence *in situ* hybridization (FISH) analysis, immunofluorescence (IF) microscopy and cytogenetic analysis

For FISH analysis, BJ cells were seeded and grown directly on coverslips, then hybridized with fluorophobe-labeled chromosome enumeration 6p11.1-q11 alpha satellite DNA FISH probe employing manufacturer's protocol and reagents (Abbott Molecular, Abbott Park, IL). For IF microscopy, BJ cells seeded and grown on coverslips, were fixed in methanol at -20°C for 30 min, then immersed rapidly in ice-cold acetone for a few seconds. The slides were allowed to dry at room temperature and rehydrated in PBS. Nonspecific binding sites were blocked in PBS containing 5% FBS (blocking solution); then, the slides were incubated sequentially with specific primary Ab (10 μg/ml in blocking solution) and specific labeled secondary Ab (10 μg/ml in blocking solution; Alexa-594 Fluor goat anti-mouse or goat anti-rabbit from Invitrogen Corp.), followed by 4′,6-diamidino-2-phenylindole (DAPI) staining, also in blocking solution.

For dual FISH/IF-labeling, FISH was performed prior to IF staining. Images were generated with a Nikon microsystem mounted on a Nikon Eclipse E600 microscope with a photometric Cool-Snap HQ2 camera and Nikon NIS-Elements software 9 (v 3.8AR) and with a Zeiss Axio Observer Z1 automated microscope and Axiovision software (v4.8.2). Images including densitometry analysis, were analysed by Image J software (v1.49), a Java-based processing program developed by the National Institutes of Health (USA). Metaphase preparation, G-banding techniques and cytogenetic analysis were performed according to standard cytogenetic procedures. Clonal chromosomal abnormalities were reported according to the recommendations of the International System for Human Cytogenetic Nomenclature (2013). Statistical analyses (student's t test) were conducted with Prism GraphPad software (v 5.0d).

## Results

### Expression of Bcl-xL phosphorylation mutants in BJ cells and effects on cell population doubling and cell fate

Studies were conducted in BJ cells expressing human influenza hemagglutinin (HA)-tagged Bcl-xL(wt), (S49A), (S49D), (S62A), (S62D) or dual (S49/62A) and (S49/62D) phosphorylation mutants. The cells were infected with lentiviruses expressing various cDNAs at early cell population doubling ranging from 25.42 to 27, as indicated on graph (*x* axis, time-point 0), and the kinetics of cell population doubling were monitored over a period of 4 months post-infection, until cell populations stop proliferating.

**Fig 1:**
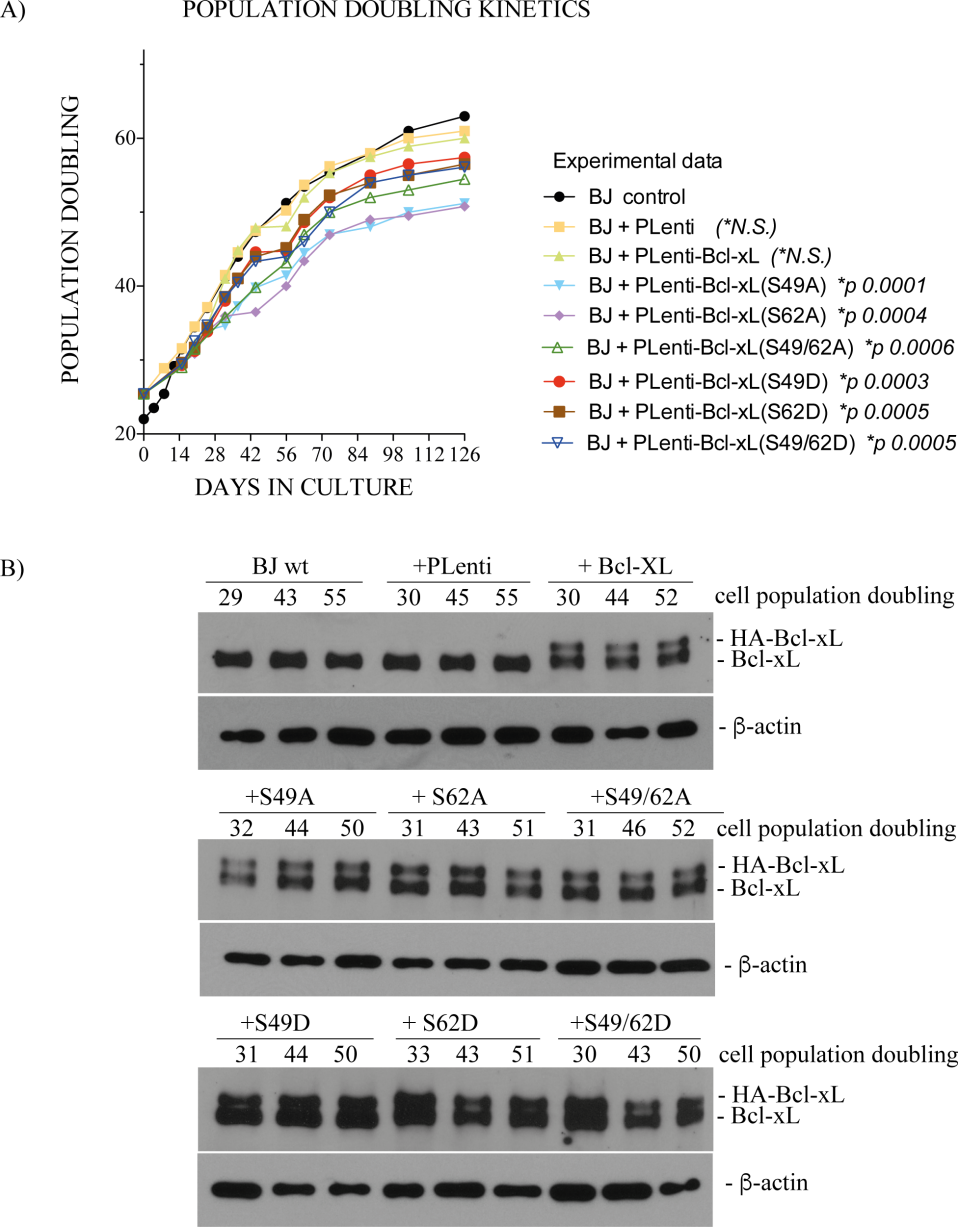
Effect of Bcl-xL and Bcl-xL phosphorylation mutant expression on cell population doubling of BJ cells. (**A**) Population doubling kinetics of control BJ cells and BJ cells expressing empty lentivirus vector or lentivirus vectors encoding HA-Bcl-xL(wt), (S49A), (S49D), (S62A), (S62D) or dual (S49/62A) and (S49/62D) phosphorylation mutants. **(B)** Expression kinetics of endogenous Bcl-xL, HA-Bcl-xL(wt) and various phosphorylation mutants at various cell population doublings; β-actin expression is shown as control. SDS-PAGE was run on 9–18% gradient gels.

Fig 1A represents a typical experiment and Fig 1B illustrates the expression of endogenous Bcl-xL, HA-Bcl-xL(wt) and various phosphorylation mutants, by Western immunoblotting, at different cell population doublings. Two similar additional experiments are reported in S1 Fig. The kinetics of cell population doubling were similar in control BJ cells and BJ cells infected by control lentivirus vector or HA-Bcl-xL(wt) (Fig 1A). In contrast, cells expressing Ser49 and/or Ser62 phosphorylation mutants showed reduced kinetics of cell population doubling (Fig 1A). Significance and *p* values are indicated on graph. The apparent differences observed in kinetics between the S/A compared to S/D mutants (Fig 1A) were not statistically significant in all experiments. Expression levels of HA-Bcl-xL and HA-Bcl-xL phosphorylation mutant proteins were stable along the experiments and slightly lower compared to endogenous Bcl-xL protein, with ratios ranging from .56 to 1.06.

**Fig 2:**
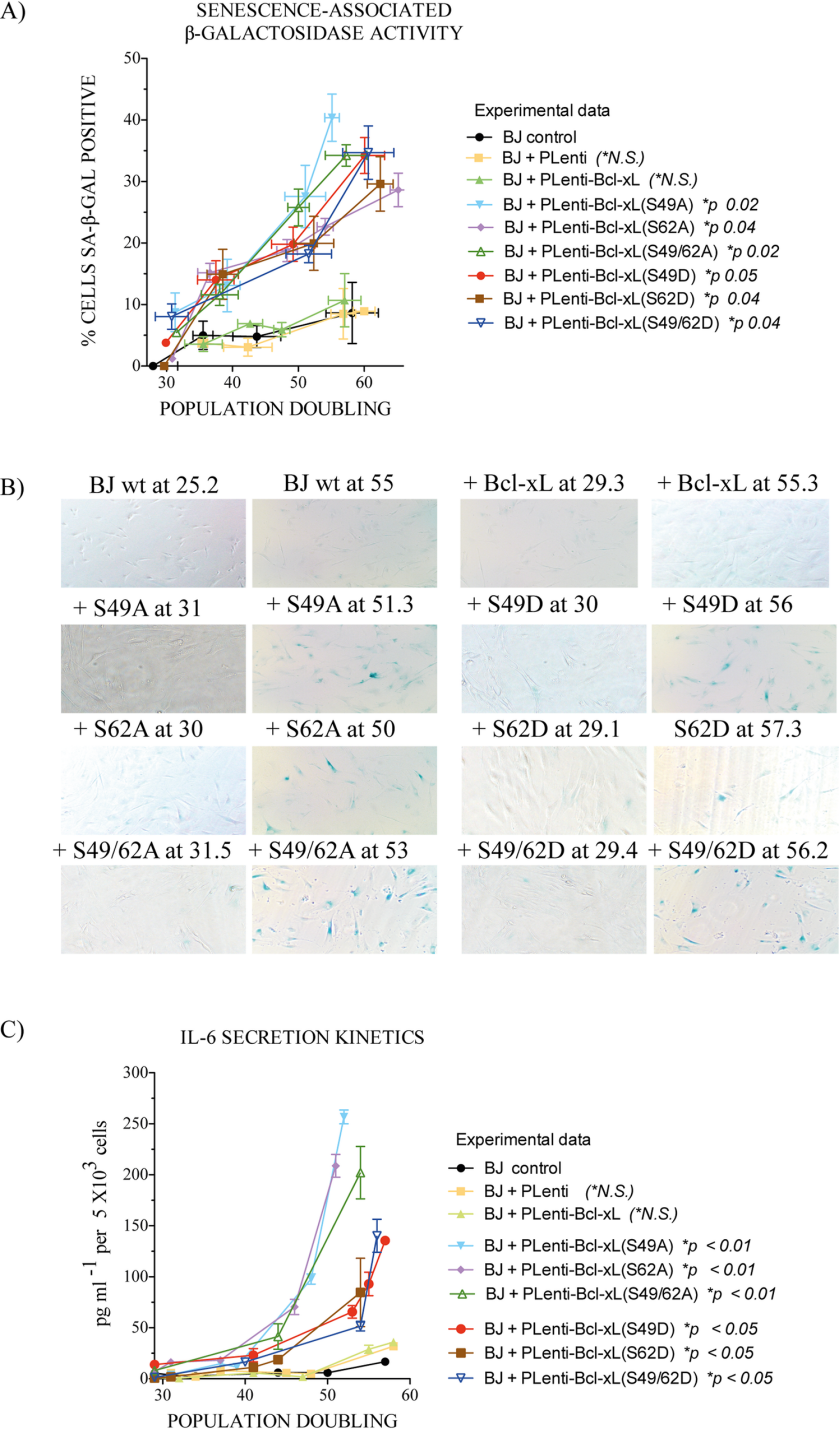
Effect of Bcl-xL and Bcl-xL phosphorylation mutant expression on outbreak of senescence in BJ cells. (**A**) Kinetics of senescence-associated β-galactosidase activity in control BJ cells and BJ cells expressing empty lentivirus vector or lentivirus vectors encoding HA-Bcl-xL(wt), (S49A), (S49D), (S62A), (S62D) or dual (S49/62A) and (S49/62D) phosphorylation mutants at various cell population doublings. The data from 3 independent experiments. **(B)** Typical micrographs of senescence-associated β-galactosidase activity in various cell populations. (**C**) Kinetics of IL-6 secretion from control BJ cells and BJ cells expressing empty lentivirus vector or lentivirus vectors encoding HA-Bcl-xL(wt), (S49A), (S49D), (S62A), (S62D) or dual (S49/62A) and (S49/62D) phosphorylation mutants at various cell population doublings (*n*: 4).

The observed decrease in the kinetics of cell population doubling was associated with increased senescence, as measured by SA-β-gal assays [30] (Fig 2A and 2B) and senescence-associated secretory phenotypes, with IL-6 secretion as biomarker [31] (Fig 2C). The apparent differences observed in IL-6 secretion between the S/A compared to S/D mutants were not statstitically significant. No significant effects on apoptotic or necrotic cell death were seen in cells expressing the HA-Bcl-xL phosphorylation mutants, with cell death rates less than 2–3% over the time-course of the experiments. The morphology of more than 25,000 Hoechst 33342- and PI-stained cells was analyzed for each phosphorylation mutant at different population doublings (data not shown).

### Expression of HA-Bcl-xL phosphorylation mutants in BJ cells and chromosome instability and aneuploidy

**Fig 3:**
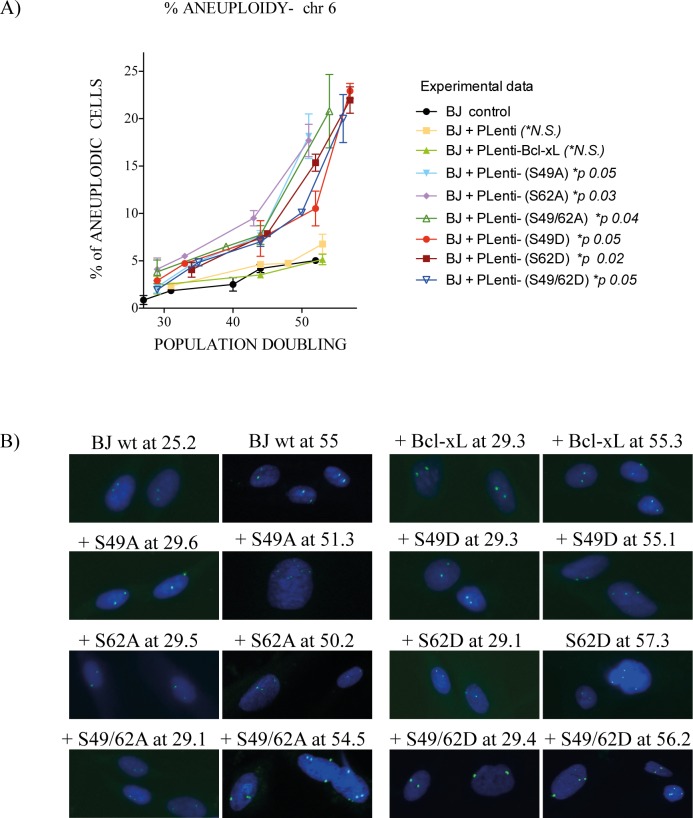
Effect of Bcl-xL and Bcl-xL phosphorylation mutant expression on chromosome stability and aneuploidy in BJ cells. (**A**) % of aneuploid kinetics in interphasic control BJ cells and BJ cells expressing empty lentivirus vector or lentivirus vectors encoding HA-Bcl-xL(wt), (S49A), (S49D), (S62A), (S62D) or dual (S49/62A) and (S49/62D) phosphorylation mutants at various cell population doublings. Total number of cells analysed: 2,639 (wt), 1,718 (S49A), 2,168 (S49D), 2,914 (S62A), 2,096 (S62D), 2,194 (S49/62A) and 2,333 (S49/62D). Micrographs from 3 to 5 independent experiments. **(B)** Typical micrographs of FISH-labeling with a fluorophobe-labeled 6p11.1-q11 alpha satellite DNA probe. G-banding karyotypes are liste in Table 1.

Strinking effects were noted by time-lapse live-cell imaging microscopy of human cancer HeLa cells expressing HA-Bcl-xL(S49A), (S62A) and dual (S49/62A) phosphorylation mutants with an increased number of cells harbouring multiple mitotic defects, including multipolar spindle, chromosome lagging and bridging, micro-, bi- or multi-nucleated cells, and cells that fail to complete mitosis [12]. To establish if the expression of Bcl-xL phosphorylation mutants in normal diploid BJ cells provokes chromosome instability and aneuplody, FISH analysis of interphasic cells was performed at various cell population doublings, with a fluorophobe-labeled 6p11.1-q11 alpha satellite DNA probe (Fig 3A). These analyses provided simple determination by looking at chromosome 6.

Fig 3A shows a significant increase of aneuploidy in all Bcl-xL phosphorylation mutants compared to control BJ cells or BJ cells infected by control lentivirus vector or HA-Bcl-xL(wt). Typical FISH micrographs are represented in Fig 3B. To validate these observations, standard cytogenetic analysis was also performed on mitotic cells at various cell population doublings. Table 1 list the various chromosomal aberrations detected and monitored by G-banded karyotyping. It is noteworthy that, typically, FISH analysis was performed on interphasic cells (either proliferative and non-proliferative or senescent cells), while G-banded karyotyping was done on metaphasic cells, which implies that these cells are proliferative, at least through 1 mitotic cycle.

**Table 1:** Chromosomal aberrations in control BJ cells and BJ cells expressing Bcl-xL (wt) and Bcl-xL phosphorylation mutants at various cell population doublings.

**Cell Population Doubling**	**Karyotypes**
**BJ control (PD 43.3)**	46,XY[22]
**BJ control (PD 70.1)**	46,XY[10]
**pLenti (PD 53.7)**	46,XY[22]
**Bcl-xL wt (PD 52.0)**	46,XY[22]
**Bcl-xL S49A (PD 42.4)**	46,XY[18]
**Bcl-xL S49A (PD 53.6)**	46,XY[20]
**Bcl-xL S62A (PD 39.2)**	46,XY,t(6;7)(q21;q32)[3]/46, XY[17]
**Bcl-xL S62A (PD 55.3)**	46,XY,add(16)p13.1[9]/48,XY,+2mar[2]/46,XY[10]
**Bcl-xL S49/62A (PD 39.0)**	46,XY[22]
**Bcl-xL S49/62A (PD 52.1)**	46,XY[7]
**Bcl-xL S49D (PD 37.1)**	46,XY,add(16)q2?2)[4]/46,XY[12]
**Bcl-xL S49D (PD 54.1)**	46,XY,i(18)(q10)[3]/46,XY[18]
**Bcl-xL S62D (PD 40.1)**	46,XY[22]
**Bcl-xL S62D (PD 52.5)**	46,XY,t(4:5)(p16:q15)[9]/47,XY,+7[3]/46,XY[5]
**Bcl-xL S49/62D (PD 50.1)**	46,XY[12]

In cells expressing Bcl-xL phosphorylation mutants S62A, S49D and S62D, chromosomal abnormalities were detected, indicating that even in the presence of chromosome abnormalities, these cells were able to undergo at least through 1 mitotic cycle (Table 1). Control BJ cells or BJ cells infected by control lentivirus vector or HA-Bcl-xL(wt) presented very low-level aneuploidy, based on FISH analysis (Fig 3A and 3B), and have normal karyotypes, based on cytogenetic analysis (Table 1).

### Expression of senescence-associated phenotypes and biomarkers in BJ cells expressing HA-Bcl-xL phosphorylation mutants

Senescent cells can display a series of phenotypes, including SA-β-gal activity [30] (Fig 2A and 2B), senescence-associated secretory phenotypes [31] (Fig 2C), as well as nuclear foci linked to chromatin alterations and activation/recruitment of DNA damage response proteins, such as phospho-histone γH2A.X [32]. Senescence is also often associated with sustained expression of the cell cycle-dependent kinase inhibitors p16/INK4A and/or p21Waf1/Cip1 [33].

**Fig 4:**
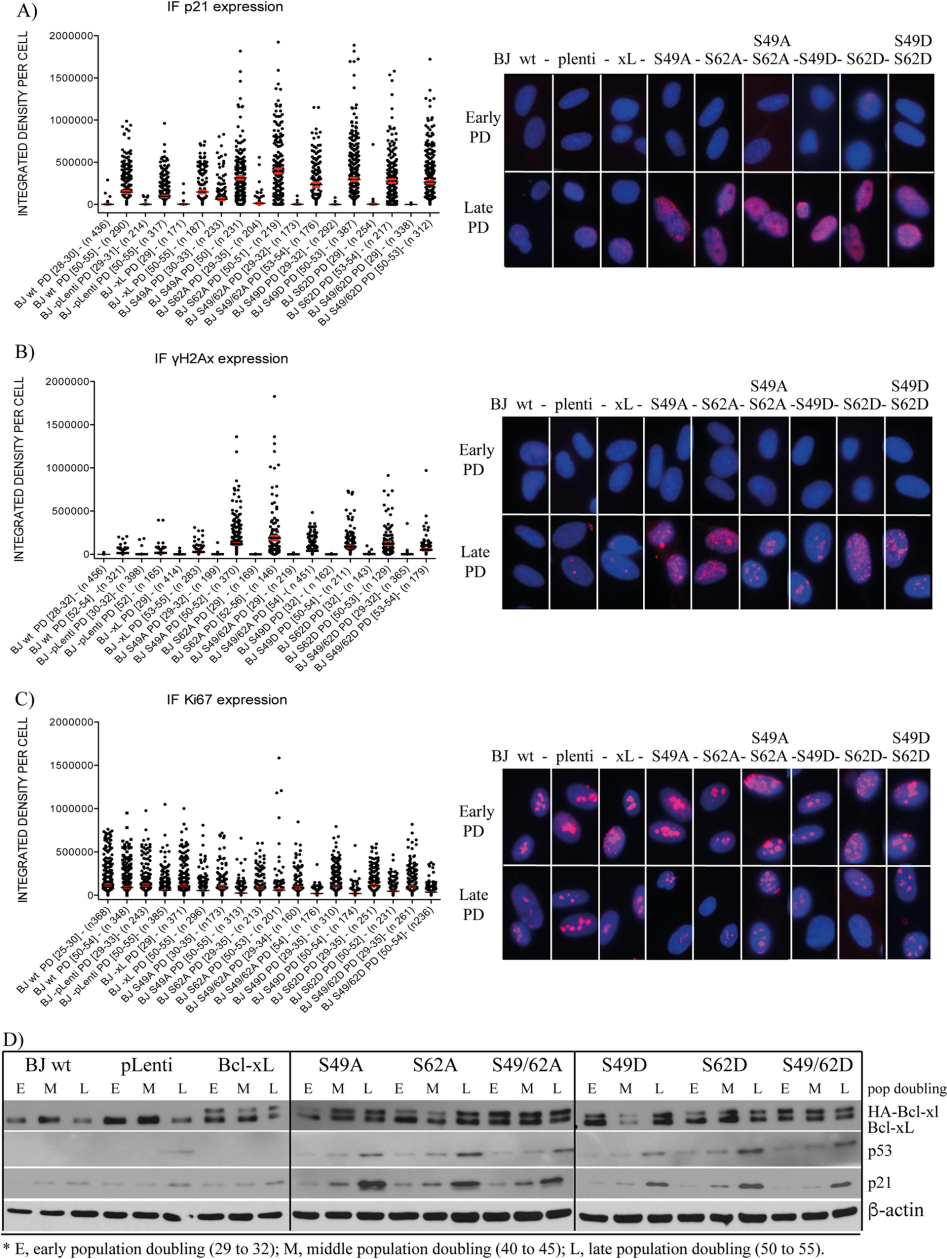
Effect of Bcl-xL and Bcl-xL phosphorylation mutant expression on senescence-associated biomarkers in BJ cells. IF-revealed expression level of **(A)** p21Waf1/Cip1, **(B)** γH2A.X, and **(C)** Ki-67 in early *versus* late population doubling of control BJ cells and BJ cells expressing empty lentivirus vector or lentivirus vectors encoding Bcl-xL(wt) and various Bcl-xL phosphorylation mutants. Left panels, X axis: The BJ cell population is indicated with population doubling range (DP [range]) and the numbers of individual cells analysed and represented in the histographs (*n*). Data were collected from a multitude of independent micrographs. Right panels: Typical micrographs showing all cell populations at early (29 to 32) and late (50 to 55) population doubling. (**D**) Expression kinetics of Bcl-xL, HA-Bcl-xL (and phosphorylation mutants), p53 and p21Waf1/Cip1 in the control BJ cell population and BJ cells expressing empty lentivirus vector or lentivirus vectors encoding Bcl-xL(wt) and the various Bcl-xL phosphorylation mutants at early (29 to 32), middle (40 to 45) and late (50 to 55) population doublings, revealed by Western blotting; β-actin expression is shown as control. SDS-PAGE was run on 9–18% gradient gels.

Fig 4A, 4B and 4C (left panels) illustrate, at the single cell level, the expression of key biomarkers revealed by immunofluorescence (IF) imaging and analysis. Both p21Waf1/Cip1 (Fig 4A) and γH2A.X (Fig 4B) expression increased significantly in late population doubling BJ cell compared to corresponding early population doubling cells. p21Waf1/Cip1 and γH2A.X expression was increased much more significantly in late population doubling BJ cells expressing Bcl-xL phosphorylation mutants compared to late population doubling control BJ cells or BJ cells infected by control lentivirus vector or HA-Bcl-xL(wt) (*p* < 0.01; not indicated on graphs). In contrast, Ki67 expression (Fig 4C), a marker of proliferative cells, decreased significantly in late population doubling BJ cells expressing Bcl-xL phosphorylation mutants compared to corresponding early population doubling cells, and population doubling control BJ cells or BJ cells infected by control lentivirus vector or HA-Bcl-xL(wt) (*p* < 0.01; not indicated on graphs). These observations are consistent with the kinetics of population doubling (Fig 1B) and the outbreak of senescence in late population doubling BJ cells expressing Bcl-xL phosphorylation mutants (Fig 2A).

Typical IF micrographs are presented in Fig 4A, 4B and 4C (right panels). Involvement of the p53 and p21Waf1/Cip1 DNA damage response pathway was confirmed by Western blottings (Fig 4D). p16/INK4A expression was barely detectable in BJ cells, even in late population doubling cells exhibiting a high senescence rate (data not shown), suggesting that p16/INK4A is not part of the process.

We attempted to correlate the expression of these biomarkers with aneuploidy at the single cell level in a limited number of samples. To do so, we implemented a FISH labeling experimental protocol, followed by IF labeling. Most, but not all cells, harbouring aneuploidy, detected by FISH, displayed high-level p21Waf1/Cip1 expression and low-level Ki67 expression (S2 Fig). These observations correlated at the single cell level, with aneuploidy, p21Waf1/Cip1 and Ki67 expression, consistent with non-proliferative and/or senescent cells. Interestingly, correlation did not fit all aneuploid cells, indicating a mosaic or progressive response, where some aneuploid cells still had proliferative potency at least for 1 or a few cell cycle divisions, finding consistent with our ability to perform G-banding analysis at metaphase (Table 1). Attempts to detect aneuploidy and γH2A.X-associated nuclear foci or senescence-associated β-galactosidase activity by similar experimental approaches were unsuccessful; the FISH experimental protocol involved an alkaline DNA denaturation step that most likely released nuclear foci-associated proteins from chromatin and destroyed acidic β-galactosidase activity (data not shown).

## Discussion

Together, our experiments revealed that the expression of Bcl-xL(S49) and (S62) phosphorylation mutants in normal human diploid BJ cells provoked chromosome instability and aneuploidy. These effects correlated with reduced cell population doubling and the outbreak of senescence with typical senescence-associated phenotypes, including high-level senescence-associated β-galactosidase activity, IL-6 secretion, p53 and p21Waf1/Cip1 expression and γH2A.X-associated nuclear foci. Our observations suggest that dynamic Bcl-xL(S49) and (S62) phosphorylation and dephosphorylation cycles are key determinants of Bcl-xL functions in maintaining chromosome integrity. These effects, by Bcl-xL(S49) and (S62) phosphorylation mutants during mitosis, are consistent with previous findings in cancer cells [10, 12]. They are also consistent with Ser49 and Ser62 which are located within the protein's unstructured loop domain [21, 22], are non-essential for Bcl-xL anti-apoptotic function [9–12], but indeed play roles in chromosome stability.

Our study revealed that, concomitant with chromosome abnormalities mediated by the expression of Bcl-xL(S49) and (S62) phosphorylation mutants, BJ cells underwent senescence. This observation further reinforced the concept that senescence can act as a potent tumor suppressing mechanism in normal cells [34]. Interestingly, Bcl-xL is very rarely mutated in human tumors, suggesting that putative key mutations within Bcl-xL would be unsuitable for cell proliferation and survival (see mutations and polymorphisms in S3 Fig). Bcl-xL overexpression rather than mutation is associated with tumor development and poor treatment response in various cancers [35–41]. Indeed, tumor cells are believed to depend on, or are addicted to, anti-apoptotic Bcl-2 family members, including Bcl-xL [42], providing a selective advantage to cancer cells by allowing them to survive various stressful environments, cell stress phenotypes and/or cell death signals that directly ensue from oncogenic signaling, tumor suppressor deficiency or anticancer treatments [42]. Although Bcl-xL(S49) and (S62) are not found yet, mutated in cancer cells, the two major protein kinases involved in Bcl-xL phosphorylation during mitosis, PLK1 and PLK3 are often linked to aneuploidy and cancer development. Indeed, human PLK1 is essential during mitosis, DNA damage responses and for maintenance of genomic stability [43]. The spatio-temporal regulation of PLK1 direct its activity at various locations, including cytoplasm, centrosomes, along microtubules, at spindle midzones, kinetochore/centromere regions and in post-mitotic bridges of the dividing cells [43]. Many studies showed the various roles of PLK1 during mitosis, more importantly, its role in ensuring SAC fidelity, kinetochore-microtubule attachment and sister chromatid separation. Misexpression of PLK1 causes mitotic abnormalities including aneuploidy leading to tumorigenesis, and its often found overexpressed in a variety of tumors [43]. PLK3 is also involved in regulating a variety of molecular and cellular events, including DNA replication, DNA damage responses, cell cycle control and tumor angiogenesis [44]. Aberrant expression of PLK3 is also found in different types of tumors [44]. Small-molecule inhibitors of PLKs are under clinical trials and provided a survival benefit for patients with leukemia [45–47]. Suppression or aberrant PLK1 and PLK3 activities would lead to defect in Bcl-xL (Ser49) and (Ser62) dynamic phosphorylation.

Our data indicate that if a putative mutation occurs randomly within Bcl-xL(S49) or (S62) in normal cells, they will undergo aneuploidy with senescence, rather than outbreak into a tumorigenesis path. However, the possibility that mutations within oncogenes or tumor suppressor genes, in combination with Bcl-xL mutations, could lead to a tumorigenesis path cannot be ruled out completely. Nevertheless, the fact that Bcl-xL mutations are very rarely found in human tumors, and yet, to the best of our knowledge, have never been detected on Ser49 and Ser62, strongly suggests that putative random mutations within Bcl-xL(S49) or (S62) in normal cells will lead to senescence outbreak. Perturbation of the SAC is well-known to result in chromosome mis-segregation and aneuploidy. Only few studies have ascertained correlations between aneuploidy and the outbreak of senescence. Reduced BubR1 expression in mouse embryonic fibroblasts causes increased aneuploidy and senescence, an effect associated with opposing roles of p16/INK4A and p19/Arf controlling senescence and aging [48, 49]. Furthermore, in mouse embryonic fibroblasts, Bub1 mutation which causes high rates of chromosome mis-segregation and aneuploidy, has been reported to be accompanied by growth defects, premature senescence, as well as tumorigenesis [50].

One of the main questions raised by this study is: how do phospho-Bcl-xL(S62) and (S49) act at the molecular level during mitosis? In a previous study, we demonstrated that phospho-Bcl-xL(S62) localizes in mitotic cytosol with some SAC signalling components, including PLK-1, BubR1 and Mad2. In addition, a series of co-immunoprecipitation experiments, on taxol- and nocodazole-exposed cells, revealed that phospho-Bcl-xL(S62) binds with Mad2-, BubR1-, Bub3- and Cdc20-complexes, but not Bub1 and Cdc27, a subunit of anaphase-promoting complex/cyclosome (APC/C) itself [12]. These interactions were confirmed by series of reciprocal co-immunoprecipitations in 2 cancer cell lines [12]. Intriguingly, when Bcl-xL is phosphorylated on Ser62, mitosis occurs normally, while expression of the non-phosphorylation mutant S62A, leads to many defects, including, delayed anaphase and chromosome mis-segregation [12]. Moreover, only the phospho-Bcl-xL(S62) form, and not the S62A form, binds to Cdc20-, Mad2-, BubR1-, Bub3-bound complexes, suggesting that it has a salutary effect on SAC resolution and proper mitosis progression [12]. Further work is ongoing to understand these protein:protein interactions and their impact on APC/C-cdc20 ubiquitin ligase activity and anaphase entry.

The molecular mechanisms of phospho-Bcl-xL(S49) action is more mysterious. Our previous observations indicate that phospho-Bcl-xL(S49) localizes at centrosomes in the G2 phase of the cell cycle and could possibly play roles in centrosome biology and microtubule elegation[10], effects that could have consequences during mitosis. During telophase/cytokinesis, phospho-Bcl-xL(S49) is found in the mid-zone body [10], a region where membrane vesicule fusion occurs, to provide the necessary membrane addition that will surround 2 daughters cells during full ingression of the contractile ring and abscission [51]. Considering that Bcl-xL has been reported to play role in membrane remodelling [4], it is tempting to speculate on phospho-Bcl-xL(S49) in the mid-zone body, promoting membrane vesicle recruitment to provide the necessary membrane addition for complete abscission of mother cells into daughters cells. These hypothesis will need to be evaluated in the near future. Similarly, the involvement of centrosome-associated phospho-Bcl-xL(S49) (late G2) and phospho-Bcl-xL(S62) (prometaphase and metaphase) in microtubule elongation and chromosome capture remains to be elucidated.

Many efforts, including new clinical trials, are currently being pursued to develop new drugs targeting the anti-apoptotic domain of Bcl-2 protein members, including Bcl-xL [52–56, 57]. In addition, recent findings, including our observations suggest that other protein activities could be of interest as targets for cancer therapy. Understanding how Bcl-xL proteins governs their mitotic functions will help to develop and explore strategies in the near future to identify novel compounds that focus not only on the anti-apoptotic domain, but also on the mitotic domain of Bcl-xL for cancer treatment.

## Supporting Information

S1 Fig Kinetics of cell population doubling of control BJ cells and BJ cells expressing empty lentivirus vector or lentivirus vectors encoding HA-Bcl-xL(wt), (S49A), (S49D), (S62A), (S62D) or dual (S49/62A) and (S49/62D) phosphorylation mutants. Two additional independents experiments are reported.
(TIF)

S2 Fig Correlation between aneuploidy and senescence-associated biomarkers in control BJ cells and BJ cells expressing Bcl-xL(wt) and Bcl-xL phosphorylation mutants. IF-revealed expression of **(A)** p21Waf1/Cip1 and **(B)** Ki-67 in late population doubling of control BJ cells and BJ cells expressing Bcl-xL(wt) or various Bcl-xL phosphorylation mutants harbouring aneuploidy on chromosome 6. Left panels *x* axis: The BJ cell population is indicated with population doubling number (DP [range]) and numbers of individual aneuploid cells detected over total number of cells observed (n). Right panels: Typical micrographs of aneuploid cells (upper panels). Controls are shown in lower panels.
(TIF)

S3 Fig Bcl-xL somatic mutations found in human tumours and short genetic variations. (TIF)
