# Supplementary figures and images for: Dynamic Bcl-xL (S49) and (S62) Phosphorylation/Dephosphorylation during Mitosis Prevents Chromosome Instability and Aneuploidy in Normal Human Diploid Fibroblasts
Source: PLoS One. 2016 Jul 11;11(7):e0159091. doi: 10.1371/journal.pone.0159091 (PMC4939973; doi:10.1371/journal.pone.0159091)

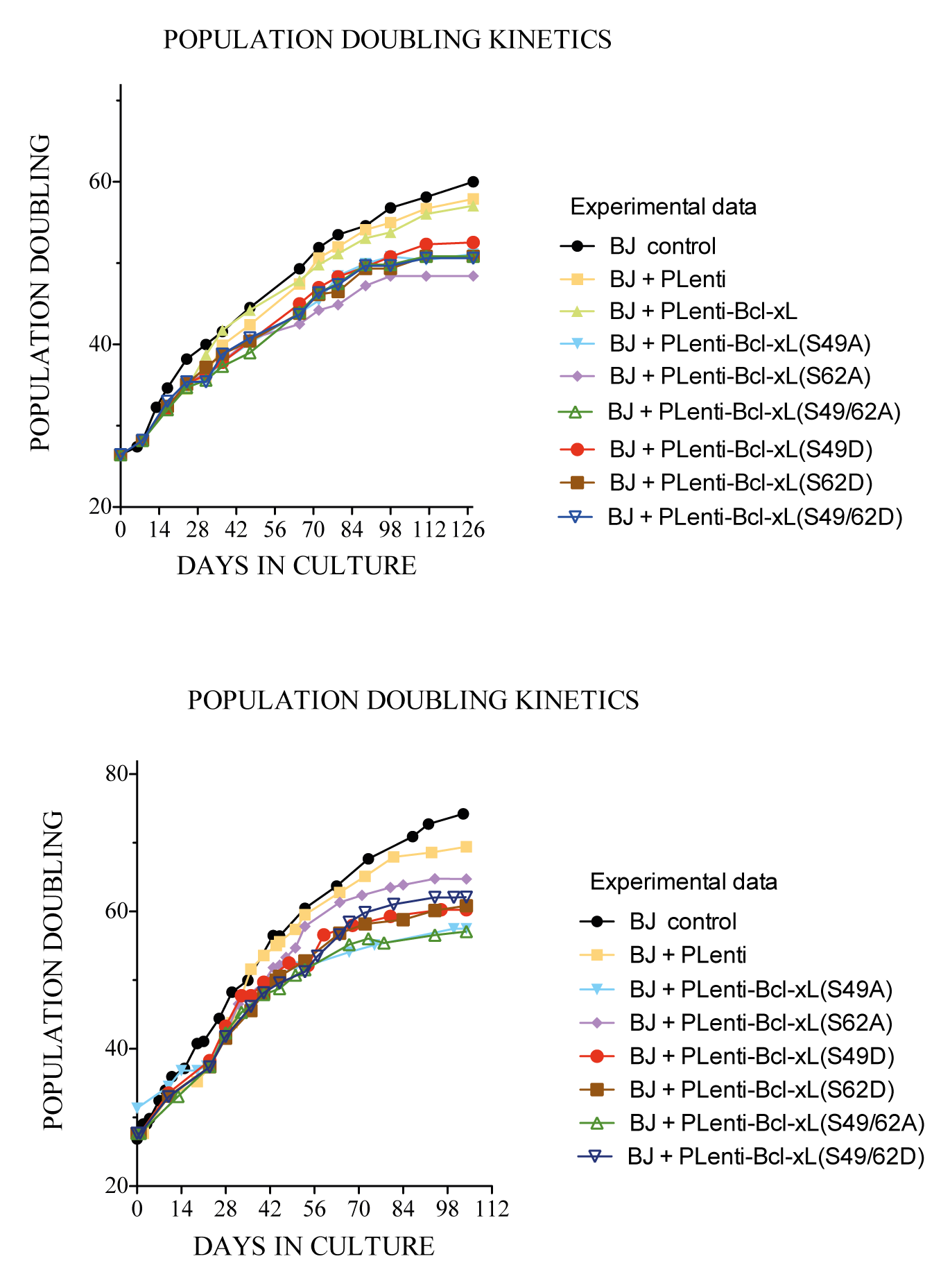

Supplement: S1 Fig — Two additional independents experiments are reported. (TIF) [file pone.0159091.s001.tif]

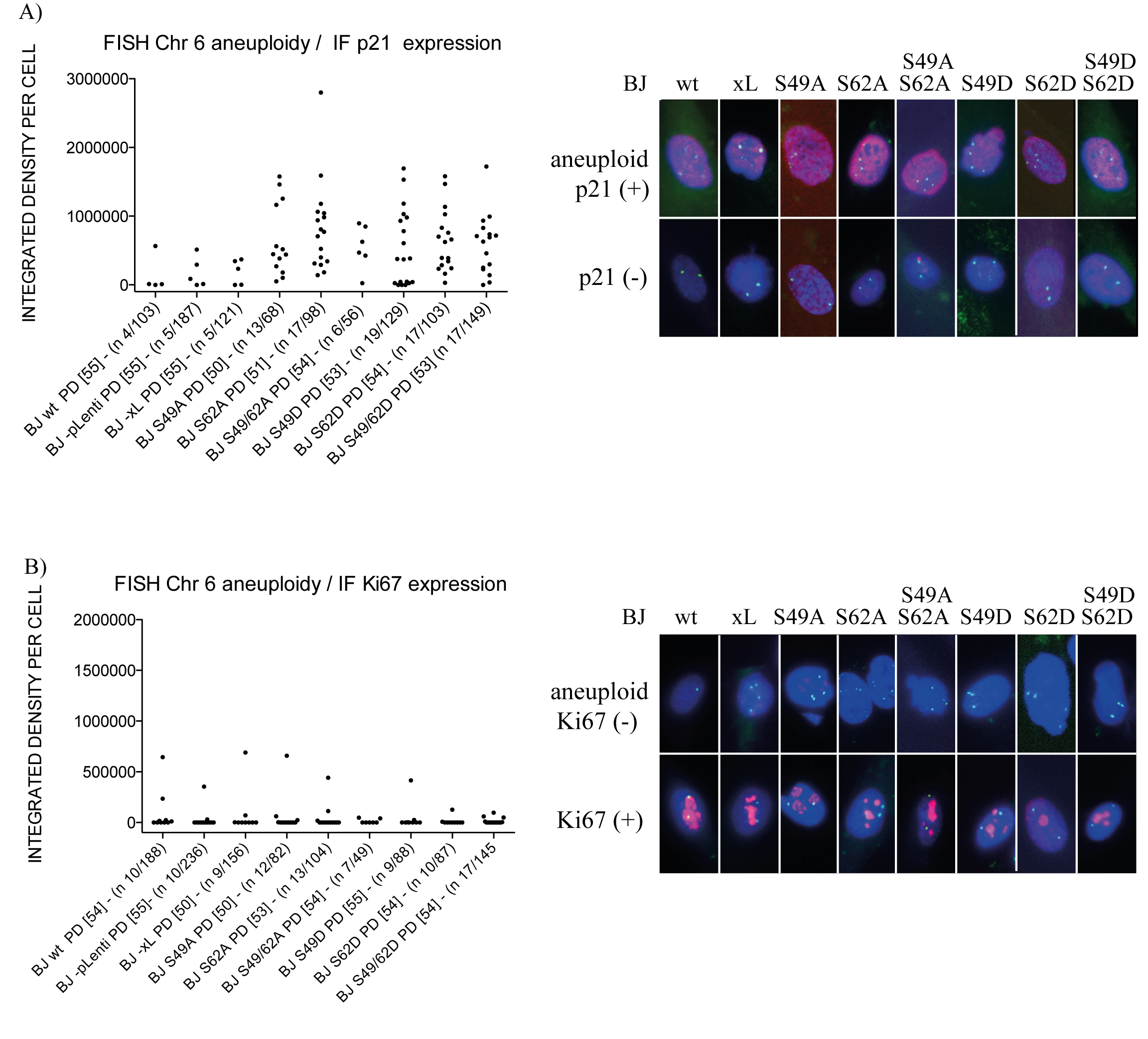

Supplement: S2 Fig — IF-revealed expression of (A) p21Waf1/Cip1 and (B) Ki-67 in late population doubling of control BJ cells and BJ cells expressing Bcl-xL(wt) or various Bcl-xL phosphorylation mutants harbouring aneuploidy on chromosome 6. Left panels x axis: The BJ cell population is indicated with population doubling number (DP [range]) and numbers of individual aneuploid cells detected over total number of cells observed (n). Right panels: Typical micrographs of aneuploid cells (upper panels). Controls are shown in lower panels. (TIF) [file pone.0159091.s002.tif]

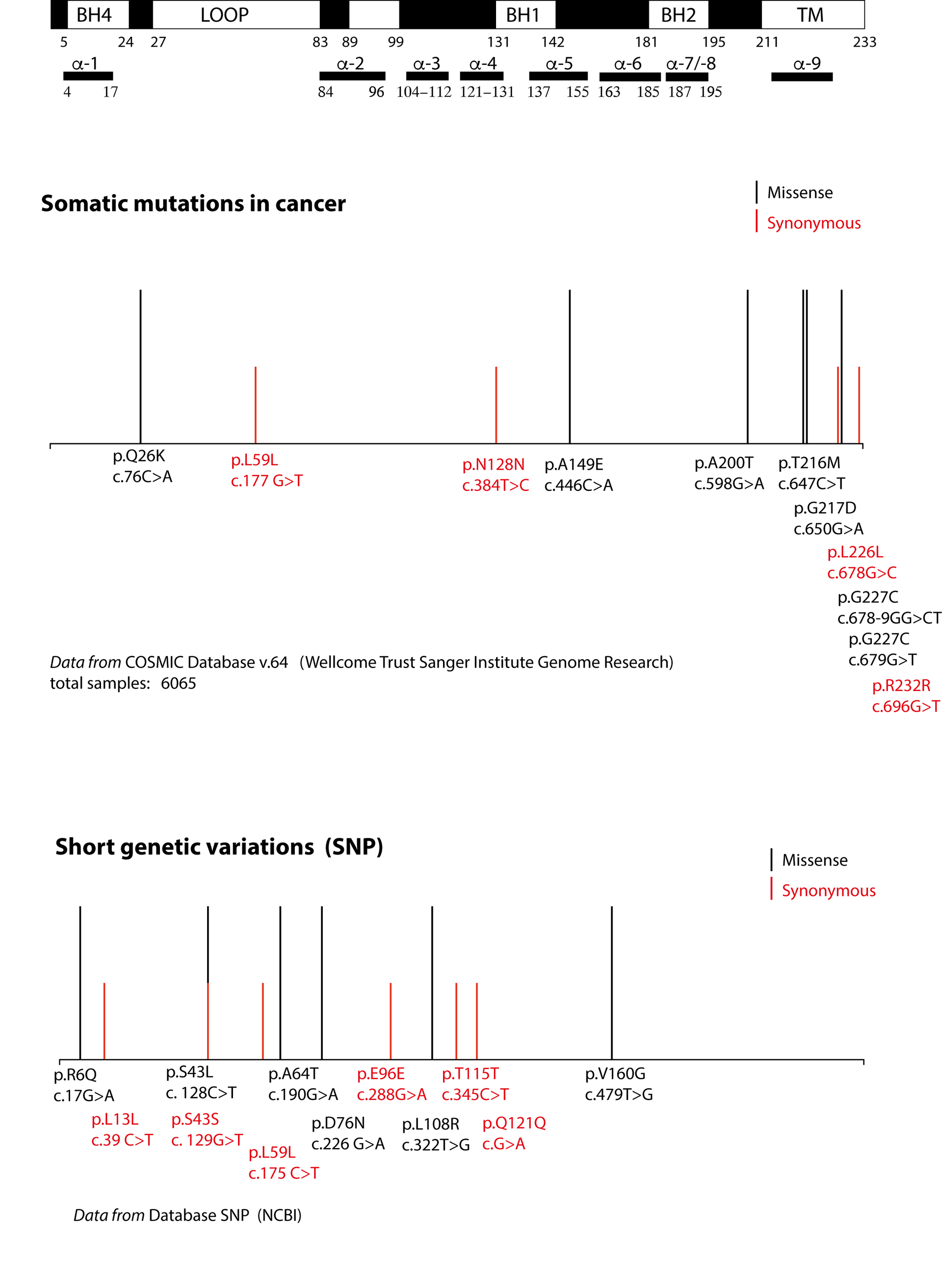

Supplement: S3 Fig — (TIF) [file pone.0159091.s003.tif]
